# Incidence and prevalence, and medication use among adults living with dermatomyositis: an Alberta, Canada population-based cohort study

**DOI:** 10.1038/s41598-023-43880-7

**Published:** 2023-09-30

**Authors:** Mohammed Osman, Karen J. B. Martins, Kai On Wong, Khanh Vu, Alexis Guigue, Jan Willem Cohen Tervaert, Robert Gniadecki, Scott W. Klarenbach

**Affiliations:** 1 Faculty of Medicine and Dentistry, Department of Medicine, University of Alberta, Edmonton, AB T6G 2R3 Canada; 2 Faculty of Medicine and Dentistry, Real World Evidence Unit, University of Alberta, Edmonton, AB T6G 2R3 Canada; 3 Cumming School of Medicine, Centre for Health Informatics, University of Calgary, Calgary, AB T2N 1N4 Canada

**Keywords:** Inflammatory diseases, Epidemiology

## Abstract

Dermatomyositis is a rare disease characterized by progressive muscle weakness and skin rashes. Estimates of incidence and prevalence are fundamental measures in epidemiology, but few studies have been conducted on dermatomyositis. To address this knowledge gap, we conducted a population-based study to determine the contemporary incidence (between 2013 and 2019) and prevalence (2019) of adults living with dermatomyositis using administrative health data in Alberta, Canada. We also described disease-related medication use, as there are very few approved medications for the treatment of dermatomyositis, and no Canadian therapeutic guidelines. The average age- and sex-standardized annual incidence of dermatomyositis was 2.8–3.0 cases per 100,000 adults, and prevalence was 28.6 cases per 100,000 adults, which is greater than reported in other cohorts. Dermatomyositis-related medication use decreased from 73% in the first year to 46% in the eighth year after diagnosis. Glucocorticoids were the most commonly used drug class, often taken concurrently with various immunomodulatory agents; this medication use aligns with empirically-based recommendations and the few therapeutic guidelines for dermatomyositis. Considering that Alberta may have one of the highest rates of dermatomyositis among adults, further research on the burden of disease is warranted for planning within the health care system.

## Introduction

Dermatomyositis (DM) is a rare form of idiopathic inflammatory myopathy characterized by progressive proximal muscle weakness and characteristic cutaneous findings^1,2^. Diagnosis is based on the presence of these clinical features complemented by laboratory investigations demonstrating elevated muscle enzymes values, imaging (e.g., magnetic resonance imaging), myositis-specific autoantibodies, and/or a muscle biopsy^3^. Other common manifestations include cardiac abnormalities, interstitial lung disease, and malignancy^1^.

Females are at higher risk of developing DM, with a reported 2:1 female-to-male ratio^4,5^, and the average age of diagnosis among adults is between 40 and 60 years^4^. Among the few epidemiological reports on DM globally, incidence and prevalence estimates vary widely with reported incidence rates ranging from 0.1 to 10 cases per 100,000 person-years, and prevalence rates ranging from 1.97 to 21.5 per 100,000 population^5–13^. To our knowledge, only one epidemiologic study has been conducted in Canada, which estimated the 2003 combined prevalence of DM and polymyositis (PM), another idiopathic inflammatory myopathy subtype, at 21.5 per 100,000 in the province of Quebec^7^. Estimates of incidence and prevalence are fundamental measures in epidemiology, and understanding the burden of disease is necessary for planning within health care systems. To address this knowledge gap, we conducted a population-based study to determine contemporary incidence and prevalence rates of adults living with DM using administrative health data in Alberta, Canada. We also described medication use among this patient population, as there are very few approved medications for the treatment of DM (none have an indication for DM in Canada), and no Canadian therapeutic guidelines.

## Results

### Cohort selection

**Figure 1:**
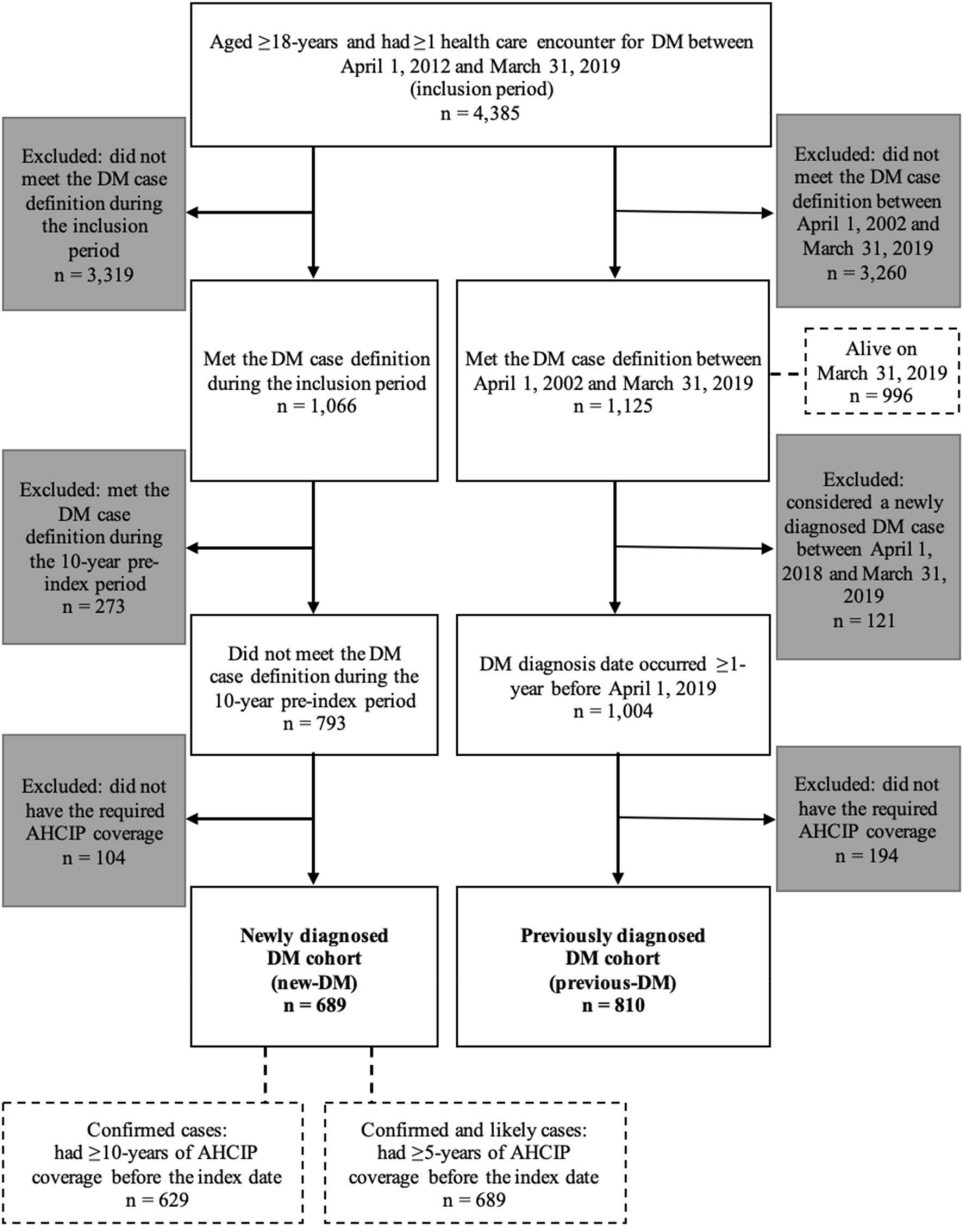
Flow diagram of cohort selection (solid boxes and arrows), and selection of incident and prevalent DM cases (dashed boxes and lines). *AHCIP* Alberta Health Care Insurance Plan, *DM* dermatomyositis.

Among the 4,385 adults (aged ≥ 18 years) with ≥ 1 recorded diagnostic code for DM during the inclusion period (April 1, 2012 and March 31, 2019), individuals were selected for two non-mutually exclusive cohorts that included those with newly diagnosed DM (*new-DM*) and previously diagnosed DM (*previous-DM*; living with DM for ≥ 1 year). A graphical representation of cohort selection and study measures is presented in Supplementary Fig. 1. Detailed in Fig. 1, the new-DM cohort included adults with ≥ 1 recorded diagnostic code for DM during the inclusion period (4,385) who met the case definition for DM during this timeframe (1,066), and had a washout period of ≥ 10 years before the incident date (the earliest health care encounter date within the case definition) where the case definition for DM was not met, along with sufficient Alberta Health Care Insurance Plan (AHCIP) coverage (n = 689). The previous-DM cohort included adults with ≥ 1 recorded diagnostic code for DM during the inclusion period (4385) who met the case definition for DM between April 1, 2002 and March 31, 2019 (1125), and had been living with DM for ≥ 1 year as of April 1, 2019 (the index date), along with sufficient AHCIP coverage (n = 810).

### Incidence and prevalence

Between April 1, 2012 and March 31, 2019, the standardized (95% confidence interval [CI]) provincial annual incidence ranged from 2.2 (1.7, 2.8) to 3.4 (2.8, 4.1) confirmed and 2.5 (2.0, 3.2) to 3.7 (3.1, 4.4) confirmed and likely cases, with an overall average of 2.8 (2.2, 3.4) confirmed and 3.0 (2.5, 3.7) confirmed and likely cases per 100,000 adults (Table 1). Of the 1125 adults who met the case definition for DM between April 1, 2002 and March 31, 2019, 996 were alive on March 31, 2019 (Fig. 1); standardized (95% CI) prevalence was 28.6 (26.9, 30.5) cases per 100,000 adults.

**Table 1:** Annual and average crude and age-/sex-standardized incidence rates and prevalence of DM per 100,000 population ≥ 18-years of age in Alberta.

	DM cases		Adult population of Alberta	Crude per 100,000		Age-/sex-standardized per 100,000			
	Confirmed	Confirmed and likely		Confirmed	Confirmed and likely	Confirmed	95% CI	Confirmed and likely	95% CI
*Annual incidence*									
2013	72	80	3,098,198	2.3	2.6	2.4	(1.9, 3.1)	2.7	(2.1, 3.3)
2014	70	79	3,179,467	2.2	2.5	2.2	(1.7, 2.8)	2.5	(2.0, 3.1)
2015	68	80	3,222,404	2.1	2.5	2.2	(1.7, 2.7)	2.5	(2.0, 3.2)
2016	112	121	3,256,459	3.4	3.7	3.4	(2.8, 4.1)	3.7	(3.1, 4.4)
2017	92	96	3,290,711	2.8	2.9	2.7	(2.2, 3.3)	2.8	(2.3, 3.5)
2018	103	112	3,337,616	3.1	3.4	3.1	(2.5, 3.8)	3.4	(2.8, 4.1)
2019	112	121	3,392,072	3.3	3.6	3.2	(2.6, 3.9)	3.5	(2.9, 4.2)
Average	89	98	3,253,847	2.8	3.0	2.8	(2.2, 3.4)	3.0	(2.5, 3.7)
*Prevalence*									
2019	996		3,392,072	29.4		28.6 (26.9, 30.5)			

*CI* confidence interval, *DM* dermatomyositis.

### Characteristics

Table 2 outlines characteristics of the DM cohorts. The mean age of new-DM was 55 (standard deviation [SD] 17) years, and previous-DM was 57 (SD 17) years. Females comprised 66% and 69% of new-DM and previous-DM, respectively, and the vast majority resided in urban areas (new-DM: 89%; previous-DM: 88%). Charlson Comorbidity Index scores ranged from none-to-mild in 84% of new-DM and 88% of previous-DM. During the 5-year pre-incident (new-DM) or pre-index period (previous-DM), cardiovascular disease and cancer were found to be common (> 10% of individuals) in both new-DM (18% and 12%, respectively) and previous-DM (17% and 12%, respectively); interstitial lung disease was identified in 5% of the new-DM cohort and 10% of the previous-DM cohort.

**Table 2:** Baseline characteristics of the DM cohorts.

	New-DM (n = 689)		Previous-DM (n = 810)	
*Demographic*				
Age				
Years, mean (SD)	55	(17)	57	(17)
Categories, n (%)				
18–24	35	(5.1)	19	(2.4)
25–29	25	(3.6)	32	(4.0)
30–34	38	(5.5)	46	(5.7)
35–39	40	(5.8)	51	(6.3)
40–44	46	(6.7)	44	(5.4)
45–49	56	(8.1)	67	(8.3)
50–54	80	(11.6)	73	(9.0)
55–59	85	(12.3)	104	(12.8)
60–64	86	(12.5)	95	(11.7)
≥ 65	198	(28.7)	279	(34.4)
Sex, n (%)				
Female	456	(66.2)	560	(69.1)
Male	233	(33.8)	250	(30.9)
Residence, n (%)				
Rural	78	(11.3)	101	(12.5)
Urban	611	(88.7)	709	(87.5)
*Clinical*				
Charlson comorbidity index				
Categories, n (%)				
0 (none)	245	(35.6)	228	(28.2)
1–2 (mild)	335	(48.6)	481	(59.4)
3–4 (moderate)	70	(10.2)	73	(9.0)
≥ 5 (severe)	39	(5.7)	28	(3.5)
Comorbidities, n (%)				
Cardiovascular disease	124	(18.0)	141	(17.4)
Cancer	82	(11.9)	99	(12.2)
Interstitial lung disease	37	(5.4)	83	(10.3)

*DM* dermatomyositis, *SD* standard deviation.

### Medication use

DM-related medication use is presented in Table 3. The proportion of adults within new-DM that received ≥ 1 dispensation for any DM-related medication decreased from 73% in the first year to 46% in the eighth year after the DM incident date. Among those with ≥ 1 dispensation, glucocorticoids were the most commonly used drug class (new-DM: 60% annual average; previous-DM: 61%), followed by immunosuppressants (new-DM: 53% annual average; previous-DM: 60%), antimalarials (new-DM: 38% annual average; previous-DM: 34%), and immunoglobulin G (new-DM: 13–19% annual average; previous-DM: 17%); no medications within the ‘other agent’ drug class (topical alitretinoin and crisaborole, and dupilumab) were dispensed. Concurrent drug class use was also common (new-DM: 52% annual average; previous-DM: 46%). The most common drugs used concurrently were glucocorticoids and the following: immunosuppressive agents (new-DM: 16–21% annual average; previous-DM: 16%), antimalarials (9–14% annual average; 8%), immunosuppressive agents and antimalarials (5–8% annual average; 5%), and immunoglobulin G (6–8% annual average; 5%); immunosuppressive agents were commonly used concurrently with the following: antimalarials (19–23% annual average; 20%), and immunoglobulin G (8–13% annual average; 8%).

**Table 3:** DM-related medication use within the newly diagnosed and previously diagnosed DM cohorts.

	New-DM								Previous-DM^a^
	Post-incident date (years)								
	1 n = 689	2 n = 664	3 n = 523	4 n = 396	5 n = 297	6 n = 193	7 n = 129	8 n = 63	n = 810
Received ≥ 1 dispensation, n (%)									
*Any DM-related medication*	*502 (72.9)*	*424 (63.9)*	*324 (62.0)*	*237 (59.9)*	*182 (61.3)*	*113 (58.6)*	*63 (48.8)*	*29 (46.0)*	*521 (64.3)*
Glucocorticoids	437 (87.1)	305 (71.9)	203 (62.7)	149 (62.9)	114 (62.6)	71 (62.8)	32 (50.8)	12 (41.4)	317 (60.8)
Prednisone	320 (63.7)	204 (48.1)	130 (40.1)	84 (35.4)	65 (35.7)	43 (38.1)	15 (23.8)	< 10	181 (34.7)
Corticosteroids, topical	240 (47.8)	157 (37.0)	102 (31.5)	78 (32.9)	57 (31.3)	33 (29.2)	18 (28.6)	< 10	177 (34.0)
Prednisone (methyl)	16 (3.2)	< 10	< 10	< 10	< 10	< 10	< 10	< 10	14 (2.7)
Immunosuppressants	322 (64.1)	271 (63.9)	193 (59.6)	141 (59.5)	98 (53.8)	61 (54.0)	30 (47.6)	15 (51.7)	312 (59.9)
Methotrexate	206 (41.0)	162 (38.2)	110 (34.0)	76 (32.1)	48 (26.4)	27 (23.9)	12 (19.0)	< 10	172 (33.0)
Mycophenolate mofetil	69 (13.7)	78 (18.4)	53 (16.4)	42 (17.7)	35 (19.2)	20 (17.7)	11 (17.5)	< 10	102 (19.6)
Azathioprine	78 (15.5)	60 (14.2)	37 (11.4)	30 (12.7)	19 (10.4)	13 (11.5)	< 10	< 10	62 (11.9)
Tacrolimus, topical	34 (6.8)	12 (2.8)	< 10	< 10	< 10	< 10	0 (0)	0 (0)	12 (2.3)
Rituximab	12 (2.4)	< 10	< 10	< 10	< 10	< 10	< 10	< 10	18 (3.5)
Antimalarials	204 (40.6)	167 (39.4)	115 (35.5)	89 (37.6)	70 (38.5)	45 (39.8)	24 (38.1)	13 (44.8)	178 (34.2)
Hydroxychloroquine	197 (39.2)	160 (37.7)	110 (34.0)	86 (36.3)	69 (37.9)	44 (38.9)	24 (38.1)	13 (44.8)	176 (33.8)
Chloroquine	13 (2.6)	10 (2.4)	< 10	< 10	< 10	< 10	0 (0)	0 (0)	< 10
Immunoglobulin G	115 (22.9)	89 (21.0)	56 (10.7)	40 (16.9)	30 (16.5)	17 (15.0)	< 10	< 10	87 (16.7)
Concurrent drug class use	394 (78.5)	289 (68.2)	171 (52.8)	118 (49.8)	86 (47.3)	53 (46.9)	23 (36.5)	11 (37.9)	240 (46.1)
G + I	203 (40.4)	117 (27.6)	58 (17.9)	33 (13.9)	26 (14.3)	14 (12.4)	< 10	< 10	81 (15.5)
I + A	119 (23.7)	107 (25.2)	70 (21.6)	53 (22.4)	38 (20.9)	24 (21.2)	11 (17.5)	< 10	105 (20.2)
G + A	96 (19.1)	57 (13.4)	28 (8.6)	17 (7.2)	13 (7.1)	10 (8.8)	< 10	< 10	43 (8.3)
G + I + A	72 (14.3)	45 (10.6)	22 (6.8)	10 (4.2)	< 10	< 10	< 10	0 (0)	28 (5.4)
G + IgG	70 (13.9)	44 (10.4)	21 (6.5)	14 (5.9)	11 (6.0)	< 10	< 10	0 (0)	25 (4.8)
I + IgG	69 (13.7)	58 (13.7)	33 (10.2)	22 (9.3)	14 (7.7)	< 10	< 10	< 10	41 (7.9)

Significant values are in italics.

*A* antimalarials, *DM* dermatomyositis, *G* glucocorticoids, *I* immunosuppressive agents, *IgG* immunoglobulin G.

^a^DM-related medication use was observed during a 1-year period (April 1, 2019 to March 31, 2020). In each year post-incident date in the new-DM cohort and in the previous-DM cohort, between 0 and 9 individuals received ≥ 1 dispensation for the glucocorticoids of hydrocortisone or prednisone, and the immunosuppressants of abatacept, cyclosporine, cyclophosphamide, etanercept, mercaptopurine, tacrolimus, or tioguanine.

## Discussion

In this administrative health data population-based study, contemporaneous incidence rates and point prevalence of adults living with DM were determined, as well as medication use in Alberta, Canada. The standardized overall average annual incidence was estimated at 2.8–3.0 cases per 100,000 adults between 2013 and 2019, and standardized prevalence was 28.6 cases per 100,000 adults on March 31, 2019, which is greater than most previous reports^5–10,12,13^. The characteristics of adults living with DM were consistent with epidemiologic findings^4,5,14^; DM was diagnosed in the middle aged, and there were twice as many adult females with DM compared with males. During the first year after the DM incident date, 27% of individuals did not receive any DM-related medications; among those who had medication use, glucocorticoids (prednisone and topical corticosteroids) were found to be the most commonly used drug class, and other common systemic therapies included methotrexate, hydroxychloroquine, mycophenolate mofetil, and immunoglobulin G which is in alignment with empirically-based recommendations and treatment guidelines for DM from other countries^15–18^. Findings from this study provide a contemporary description of the epidemiology and medication use of adults living with DM in Alberta, Canada.

The reported incidence and prevalence of DM varies widely due to the limited number of studies conducted in this area, the different methodological approaches, and the rare nature of the disease. Reported incidence ranges from 0.1 to 10 cases per 100,000 person-year and prevalence ranges from 1.97 to 21.5 per 100,000 population^5–13^. Large population-based cohort studies have reported a DM incidence of 1.4 (2003–2008) to 1.7 (2004–2008) cases per 100,000 person-years in the USA^12,13^, and 6 to 10 cases per 100,000 person-years was estimated in Norway between 2003 and 2012^11^. To our knowledge, this study is the first to report the incidence of DM in Canada, and results indicate that incidence among the adult population in Alberta may be higher than the USA, but lower than Norway.

Applying a similar administrative-based case finding algorithm as Bernatsky et al.^7^ who identified the combined prevalence of DM/PM in Quebec, Canada at 21.5 per 100,000 individuals in 2003, we found a somewhat higher prevalence of DM in Alberta, Canada; Smoyer-Tomic et al.^13^ also applied a similar algorithm to that used by Bernatsky et al.^7^ and estimated the prevalence of DM to be lower in the USA at 9.2 cases per 100,000 in 2008. Variations in the case definition (including/not including codes for PM or the specialist type included the algorithm [rheumatologists only, or also including dermatologists and internists]), the timeframe over which the case definition was applied (Bernatsky et al. used a 14 year period from 1989 to 2003, we used a 17 year period from 2002 to 2019, and Smoyer-Tomic et al. used a 4 year period from 2004 to 2008), and the administrative databases used (Bernatsky et al. and our study utilized databases from publicly funded health care systems in Canada that cover all residents of a province, and Smoyer-Tomic et al. utilized a commercial database for those covered under private insurance plans in the USA, along with Medicaid and Medicare data from federal and state programs that cover those ≥ 65 years, some < 65 with certain conditions, and some individuals with limited income and resources) may have contributed to these varying results^7,13^. Geographical variation of the disease may also exist. Dobloug et al.^11^ found that although the overall average combined prevalence of DM/PM was 8.7 cases per 100,000 individuals in 2012 in southeast Norway, the counties included in the study varied from 6.4 to 16.4 per 100,000. Discrepancies in access to specialist care may also play a role. Bernatsky et al.^7^ found that a greater proportion of individuals living in rural areas met the case definition for DM/PM from hospitalization data compared with urban areas, and Dobloug et al.^11^ proposed that the varying density of rheumatologists across southeast Norway may have been a contributing factor to the wide variation in prevalence observed across the counties^7,11^. Collectively, it appears that a number of factors may be contributing to the wide variation in reported prevalence of DM.

In the current study, 27% of adults living with DM did not receive any disease-related medication during the first year after the incident date. This is of clinical importance as delayed initiation of appropriate treatment following onset of DM symptoms has often been reported to be an indicator of poor outcome^19^, although not in all cases^20^. Considering that we did not have clinical evaluative information in this study to confirm diagnosis date, it is possible that these individuals had not yet received a confirmed diagnosis of DM and/or were receiving treatment for a different condition. Diagnostic delay of idiopathic inflammatory myopathies has been reported, with a median delay of 15.5 months for diagnosis of DM^21,22^; a number of factors have been shown to contribute to this delay including complexity of clinical features, misdiagnoses, and incorrect treatment^22^. As with all retrospective administrative data based research, it is also possible that the individuals who did not receive a DM-related medication in this study were misidentified having DM and included in the cohort. With that said, an established case definition was used to identify adults living with DM (adapted from^7,23^) that reported a high sensitivity (88.4%, 95% CI: 75.5, 94.9) and specificity (96.4%, 95% CI: 94.9, 97.5).

While determining optimal pharmacotherapy for DM is challenging given the small number and varying outcome measures of randomized controlled trials, guidelines recommend glucocorticoids as first-line therapy^15–18^. In this study, glucocorticoids were the most commonly used drug class, and similar findings have been reported for those living with DM/PM^24^. Considering that large numbers of individuals may develop complications associated with long-term use of glucocorticoids, additional immunomodulatory agents are often utilized^1,25,26^. Studies indicate that concomitant treatment with steroid-sparing drugs reduces the glucocorticoid dose required for remission induction, lessens the relapse risk during glucocorticoid tapering, and decreases the adverse effects of glucocorticoids^3,27^. Concomitant drug use was common in this study, and four of the six most used concurrent drug class combinations included glucocorticoids with various immunomodulatory agents.

An important strength of this study is the large population-based design. However, this study is also subject to several limitations. While an established case definition was used to identify adults living with DM (adapted from^7,23^), retrospective administrative claims-based studies use data as opposed to medical records, and therefore there is a potential for misclassification of the study cohorts or outcomes. The different clinical and pathological phenotypes DM cannot be comprehensively identified using administrative data, and were therefore not reported. The Pharmaceutical Information Network (PIN) and Laboratory Information System (LIS) databases only provide information on prescription medication dispensations, and therefore may not represent actual medication uptake by individuals. Additionally, it is not known whether DM-related medications were being taken specifically for DM or for other conditions. Use of over-the-counter supplements and/or medications, prescription medications provided in a hospital or secondary care setting, and other non-pharmacotherapy management were not captured within the administrative data, and therefore not reported.

Findings from this study provide a contemporary description of the epidemiology and medication treatment patterns of adults living with DM in Alberta, Canada. Considering that Alberta may have one of the highest rates of DM among adults, further research on the burden of disease is warranted for planning within the health care system.

## Methods

The institutional review board at the University of Alberta approved this study (Pro00101589). All research was performed in accordance with relevant guidelines and regulations. Since this study only used retrospective administrative data without any direct intervention or personal identifiable information, no study participants were placed at risk; informed consent was waived by the institutional review board at the University of Alberta (Pro00101589). This study is reported according to the Strengthening the Reporting of Observational Studies in Epidemiology (STROBE) guidelines^28^.

### Study design

A retrospective observational cohort study design was used to describe contemporary incidence rates between April 1, 2012 and March 31, 2019 and point prevalence on March 31, 2019, as well as medication use between April 1, 2012 and March 31, 2020 among adults living with DM using province-wide administrative health data in Alberta, Canada from April 1, 2002 to March 31, 2020.

### Data source

Administrative data was linked to the Population Registry, which contains demographic information for all individuals in Alberta with AHCIP coverage, of which over 99% of the Alberta population participates^29^. Hospital admissions were obtained from the Discharge Abstract Database that contains primary and secondary diagnostic codes using the International Classification of Disease—Version 10—Canadian Enhancement (ICD-10-CA). Practitioner Claims includes information on fee-for-service, alternative payment plan physician billing, and shadow billing; up to 3 ICD—Version 9—Clinical Modification (ICD-9-CM; Alberta specific) diagnostic codes can be used per visit. PIN contains information on dispensed prescription medications from community pharmacies, and immunoglobulin G dispensations were obtained from LIS.

### Cohort selection

Adults with ≥ 1 health care encounter for DM (ICD-9-CM 710.3 or ICD-10-CA M33.1 located in any diagnostic field) between April 1, 2012 and March 31, 2019 (inclusion period) were considered for the non-mutually exclusive newly diagnosed and previously diagnosed DM cohorts. From these individuals, the new-DM cohort was defined as those who (1) met the following case definition for DM between April 1, 2012 and March 31, 2019 (inclusion period): ≥ 1 practitioner claim for DM (710.3) by a rheumatologist, dermatologist, or internist OR ≥ 2 practitioner claims for DM by any other type of physician ≥ 2 months apart but within ≤ 2 years OR ≥ 1 hospitalization with a recorded diagnosis of DM (M33.1 located in any diagnostic field) (adapted from^7,23^), and (2) did not meet the case definition for DM during the 10-year period before the incident date (the earliest health care encounter date within the case definition), and (3) had ACHIP coverage for ≥ 5-years before and ≥ 1-year after the incident date. Adults who had been living with DM for ≥ 1 year as of April 1, 2019 were included in the previous-DM cohort, as follows: (1) met the DM case definition between April 1, 2002 and March 31, 2019, and (2) the DM incident date occurred ≥ 1-year before April 1, 2019, and (3) had ACHIP coverage for ≥ 5-years before and ≥ 1-year after April 1, 2019; the index date was April 1, 2019. A graphical representation of new-DM and previous-DM cohort selection and study measures is presented in Supplementary Fig. 1.

### Study measures

From new-DM, incidence among the adult population of Alberta was calculated from dividing the annual number of new DM cases between April 1, 2012 and March 31, 2019 by the associated annual population of adults in Alberta during this time (obtained from the Government of Alberta)^30^. The annual number of new DM cases was presented as a range between those with ≥ 10-years of AHCIP coverage before the incident date (confirmed new cases) and those with ≥ 5-years of coverage (confirmed and likely new cases). Prevalence was calculated based on those who met the case definition for DM between April 1, 2002 and March 31, 2019, had ≥ 1 health care encounter for DM during the inclusion period, and were alive on March 31, 2019; identified cases were divided by the adult population of Alberta in 2019 (obtained from the Government of Alberta)^30^. Results were standardized to the 2001 Canadian Census using the direct method to allow for comparison with different data sources, and presented with 95% confidence intervals (CI).

Demographic characteristics of the DM cohorts included age, sex, urban or rural residence (based on the second character of the postal code) on their respective incident (new-DM) and index dates (April 1, 2019; previous-DM)^31^. A Charlson Comorbidity Index score was determined during the 2-year pre-incident/pre-index period using ICD codes of 17 different specific medical conditions, which were weighted according to their potential for influencing mortality^32^. The specific DM-related comorbidities of cancer, cardiovascular disease, and interstitial lung disease were identified during the 5-year pre-incident/pre-index period^33–37^; see Supplementary Table S1 for details.

Medications used to treat DM were categorized into drug classes that included glucocorticoids, immunosuppressants, antimalarials, immunoglobulin G, and other agents (i.e., topical alitretinoin and crisaborole that have been used as adjunct therapies, and dupilumab that has been used to treat refractory DM^38^); see Supplementary Table S2 for details. Medication use was reported according to the proportion of individuals who received ≥ 1 dispensation for a prescription medication annually between the first and the eighth year after the DM incident date in new-DM (up to March 31, 2020 or when AHCIP coverage ended, which ever occurred earlier), and over a 1-year observation period (April 1, 2019 to March 31, 2020) in previous-DM. Medications were considered to be used concurrently when days of supply were overlapping for ≥ 30 consecutive days.

### Statistical analyses

Descriptive statistics were reported as means and SD or counts and percentages, where appropriate. In accordance with data custodian privacy standards, outcomes with one to nine individuals are reported as < 10. Statistical analyses were performed using Python version 3.6.5, R version 4.1.1, and Statistical Analysis Software (SAS) 9.4 software.

### Supplementary Information

Supplementary Information.

## Data Availability

The data that support the findings of this study are available from Alberta Health Services and Alberta Health, but restrictions apply to the availability of these data, which were used under license for the current study, and so are not publicly available. Data are however available from the authors upon reasonable request and with permission of Alberta Health Services and Alberta Health.
