# Supplementary material for: Incidence and prevalence, and medication use among adults living with dermatomyositis: an Alberta, Canada population-based cohort study
Source: Sci Rep. 2023 Sep 30;13:16444. doi: 10.1038/s41598-023-43880-7 (PMC10542346; doi:10.1038/s41598-023-43880-7)
Supplement: Supplementary file 1 — Supplementary Information. [file 41598_2023_43880_MOESM1_ESM.pdf]

Supplementary Table S1. Case definitions and codes used to identify comorbidities during the 5-year pre-diagnosis / pre-index period.

Comorbidity	Algorithm	ICD-9-CM codes	ICD-10-CA codes	Other codes
Cancer	1 hospitalization or 1 ambulatory care visit or 1 claim in any years	140-165, 170-172, 174-176, 179-195, 200-208, 238.6	C00-C26, C30-C34, C37-C41, C43, C45-C58, C60-C76, C81-C85, C88, C90-C97	
Cardiovascular disease (any of the below)				
Atrial fibrillation	1 hospitalization or 2 claims in ≤2 years	427.3	I48.0	
Chronic heart failure	1 hospitalization or 2 claims in ≤2 years	398.9, 402, 404, 425.4 – 425.9, 428	I09.9, I25.5, I42.0, I42.5–I42.9, I43, I50	
Coronary artery disease	1 hospitalization or 1 ambulatory care visit or 1 procedure or 2 claims in ≤2 years	410-413	I20–I25	*Procedure codes:* 1.IJ.57.GQ, 1.IJ.50, 1.IL.35, 1.IJ.76
Peripheral artery disease	1 hospitalization or 1 ambulatory care visit or 1 claim in any years	440.2	I70.2	
Stroke	1 most responsible stroke hospitalization or emergency department OR 1 other diagnosis stroke and 1 most responsible z-code hospitalization or ambulatory care* in any years		G45 (excluding subcode G45.4), H34.0, H34.1, I60, I61, I62.9, I63, I64, I67.6; Z50 (excluding subcodes Z50.2, Z50.3, Z50.4), Z54.8, Z54.9, *only Z51.5 applies to ambulatory care	
Interstitial lung disease	2 codes >30-days apart from a hospitalization, ambulatory care visit, or physician visit	515, 516.3, 516.8, 516.9	J84.1, J84.8, J84.9	

Abbreviations: ICD-9-CM = International Classification of Disease – Version 9 – Clinical Modification; ICD-10-CA = International Classification of Disease – Version 10 – Canadian Enhancement.

Supplementary Table S2. Dermatomyositis-related medications.

Drug class	Drug name	ATC code
Glucocorticoids	prednisone	H02AB07
	(methyl) prednisone	H02AB04
	prednisolone	H02AB06
	hydrocortisone	H02AB09
	corticosteroids, topical	D07
Immunosuppressants	azathioprine	L04AX01
	methotrexate	L04AX03
	tacrolimus	L04AD02
	tacrolimus, topical	D11AH01
	cyclosporine	L04AD01
	abatacept	L04AA24
	mycophenolate mofetil	L04AA06
	etanercept	L04AB01
	cyclophosphamide (alkylating)	L01AA01
	methotrexate (antimetabolite)	L01BA01
	mercaptopurine (antimetabolite)	L01BB02
	tioguanine (antimetabolite)	L01BB03
	rituximab (monoclonal antibody)	L01XC02
Antimalarials	chloroquine	P01BA01
	hydroxychloroquine	P01BA02
Other agents	alitretinoin	D11AH04
	dupilumab	D11AH05
	crisaborole, topical	D11AH06
Immunoglobulin G	Gammagard Liquid, Gammagard S/D, Gamunex, Igivnex, IV Immune Globulin, Iveegam Immuno, Ivigex, Octagam, Panzyga, Privigen, Cuvitru, Hizentra, SC Immune Globulin	

Drugs were identified by ATC codes within the Pharmaceutical Information Network database, with the exception of immunoglobulin G therapeutic products that were identified from the Laboratory Information System by drug name. Abbreviations: ATC = anatomical therapeutic chemical.

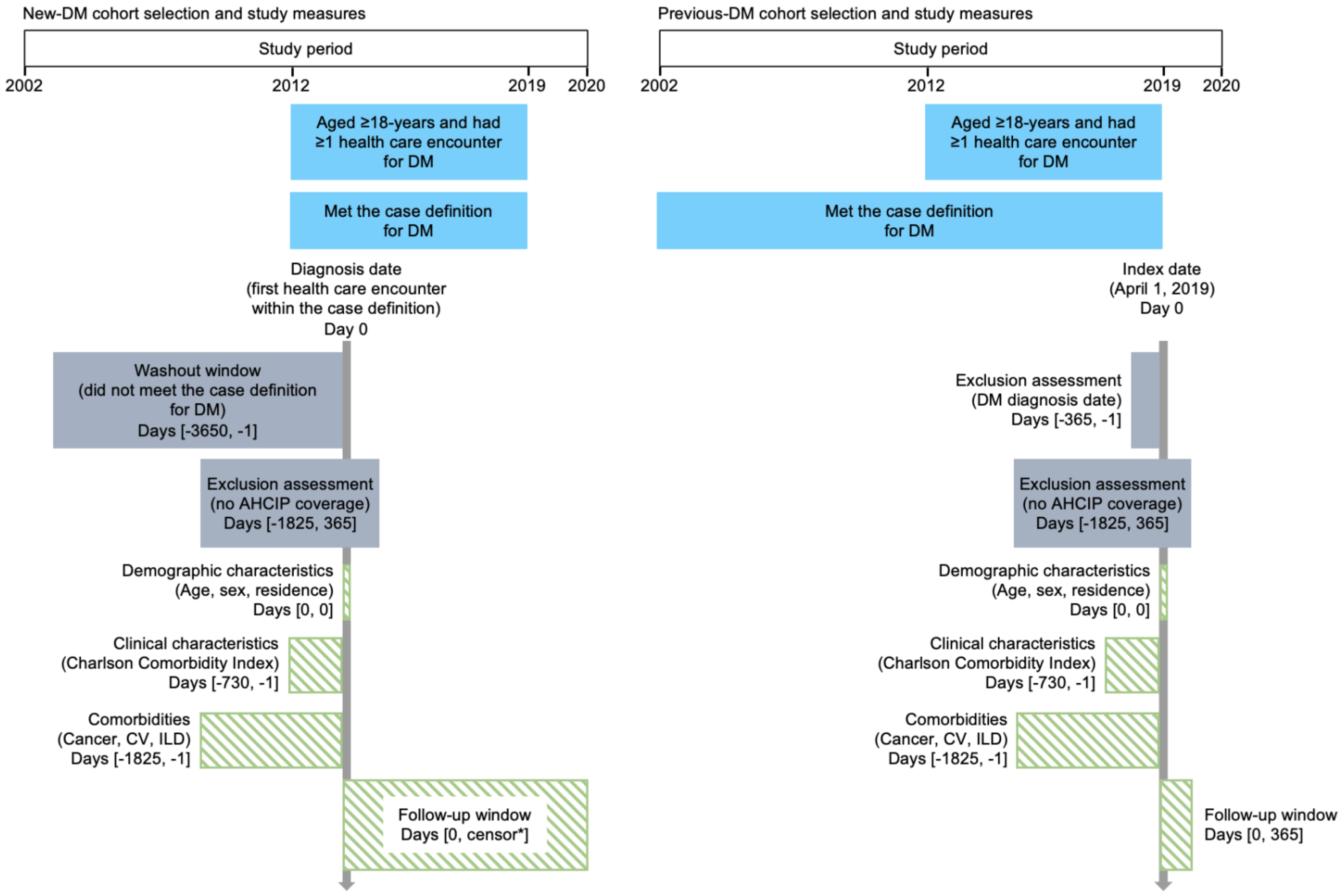

Supplementary Figure 1. Graphical representation of new-DM and previous-DM cohort selection and study measures. *Up to March 31, 2020 or when Alberta Health Care Insurance Plan coverage ended, which ever occurred earlier. Abbreviations: AHCIP = Alberta Health Care Insurance Plan; CV = cardiovascular disease; DM = dermatomyositis; ILD = interstitial lung disease.
